# OpenPheno: an open-access, user-friendly, and smartphone-based software platform for instant plant phenotyping

**DOI:** 10.1186/s13007-025-01395-4

**Published:** 2025-06-02

**Authors:** Tianqi Hu, Peng Shen, Yongshuai Zhang, Jiafei Zhang, Xin Li, Chuanzhen Xia, Ping Liu, Hao Lu, Tingting Wu, Zhiguo Han

**Affiliations:** 1 National Key Laboratory of Multispectral Information Intelligent Processing Technology, School of Artificial Intelligence and Automation, Huazhong University of Science and Technology, Wuhan, 430073 China; 2 PhenoTrait Technology Co., Ltd., Beijing, 100096 China; 3 College of Information Engineering, Northwest A&F University, Yangling, 712100 China; 4 MetaPheno Laboratory, Shanghai, 201114 China; 5 SpeCloud Technology Co., Ltd., Sanya, 572025 China; 6 Key Laboratory of Wheat Improvement, College of Mechanical and Electronic Engineering, Shandong Agricultural University, Tai’an, 271018 China; 7 College of Mechanical and Electronic Engineering, Northwest A&F University, 712100 Yangling, China

**Keywords:** OpenPheno, Plant phenotyping, Smartphone-based platform

## Abstract

**Background:**

Plant phenotyping has become increasingly important for advancing plant science, agriculture, and biotechnology. Classic manual methods are labor-intensive and time-consuming, while existing computational tools often require advanced coding skills, high-performance hardware, or PC-based environments, making them inaccessible to non-experts, to resource-constrained users, and to field technicians.

**Results:**

To respond to these challenges, we introduce OpenPheno, an open-access, user-friendly, and smartphone-based platform encapsulated within a WeChat Mini-Program for instant plant phenotyping. The platform is designed for ease of use, enabling users to phenotype plant traits quickly and efficiently with only a smartphone at hand. We currently instantiate the use of the platform with tools such as SeedPheno, WheatHeadPheno, LeafAnglePheno, SpikeletPheno, CanopyPheno, TomatoPheno, and CornPheno; each offering specific functionalities such as seed size and count analysis, wheat head detection, leaf angle measurement, spikelet counting, canopy structure analysis, and tomato fruit measurement. In particular, OpenPheno allows developers to contribute new algorithmic tools, further expanding its capabilities to continuously facilitate the plant phenotyping community.

**Conclusions:**

By leveraging cloud computing and a widely accessible interface, OpenPheno democratizes plant phenotyping, making advanced tools available to a broader audience, including plant scientists, breeders, and even amateurs. It can function as a role in AI-driven breeding by providing the necessary data for genotype-phenotype analysis, thereby accelerating breeding programs. Its integration with smartphones also positions OpenPheno as a powerful tool in the growing field of mobile-based agricultural technologies, paving the way for more efficient, scalable, and accessible agricultural research and breeding.

## Introduction

**Fig. 1:**
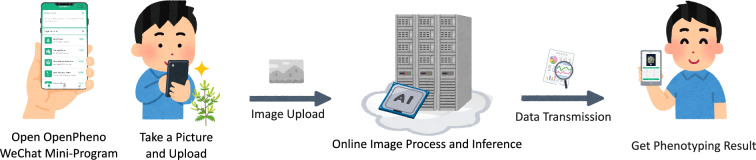
Overview of OpenPheno. OpenPheno is characterized by capturing images via a smartphone, processing data in the cloud server, and delivering real-time analysis

Plant phenotyping is crucial for plant science, agriculture, and biotechnology, forming the basis for understanding plant growth, development, and adaptation [18]. Quantifying traits such as height, leaf area, root architecture, and seed morphology allows researchers to identify and select desirable traits effectively, which is especially important for breeders. During breeding, phenotyping identifies genotypes with superior performance in specific environments.

Traditionally, plant scientists collect phenotyping traits manually, often resulting in significant time and labor costs. To overcome these challenges, computational tools have been developed to automate and standardize phenotypic measurements [10] (Table 1). From general-purpose computer vision frameworks to domain-specific solutions, these tools enhance efficiency and accuracy in phenotype analysis.

**Table 1:** Related works comparison

Tool name	Hardware device	Functionality	Features
PlantCV [7]	PC (Python)	Analyzing various plant traits	Modular architecture, code-based operation, multi-species support
HTPheno [9]	PC (ImageJ)	Measuring plant growth traits	Support for multiple species
Rosette Tracker [5]	PC (ImageJ)	Tracking rosette development	Arabidopsis-specific phenotyping
SmartRoot [15]	PC (ImageJ)	Analyzing root system architecture	Semi-automatic workflow, root-focused analysis
Phiv-Rootcell [11]	PC (ImageJ)	Quantifying rice root anatomy	Supervised analysis, rice-specific phenotyping
GRABSEEDS [24]	PC (Python)	Extracting seed, flower, and leaf traits	Command-line interface, high resource demand, multi-organ support
BerryPortraits [14]	PC (Python)	Assessing berry ripeness	Command-line interface, high resource demand, berry-specific analysis
YOLOrot2.0 [29]	PC (Python)	Measuring rice seed traits	Code-based execution, high resource demand, rice-specific analysis
PocketPlant3D [4]	Smartphone (App)	Capturing canopy architecture	Sensor-driven measurements, support for multiple species
Plant Screen Mobile [17]	Smartphone (App)	Quantifying plant projection traits	High accessibility, support for multiple species
OpenPheno (Ours)	Smartphone (WeChat)	Performing smartphone-based phenotyping with cloud processing	User-friendly interface, diverse functionality, community-driven platform, cross-platform usage

In the field of image processing, tools such as OpenCV and ImageJ [1, 20] have become indispensable. OpenCV serves developers, while ImageJ caters to advanced researchers. Both support varied image analysis tasks. PlantCV [7], an extension, adds plant-specific functions but demands significant coding skills. Consequently, these tools are mainly integrated into large-scale trait extraction workflows.

Building on foundational tools, various single-task phenotyping solutions have emerged, targeting specific analytical needs. HTPheno [9] and Rosette Tracker [5] focus on whole-plant analysis, measuring traits such as height, width, and projected shoot area. SmartRoot [15] and Phiv-Rootcell [11] specialize in root system analysis. GRABSEEDS [24], BerryPortraits [14], and YOLOrot2.0 [29] assess seed and fruit traits such as size, shape, and color. Most tools operate as ImageJ plugins or Python scripts in a PC-based environment, which limits portability. In addition, BerryPortraits and YOLOrot2.0 incorporate deep learning, requires substantial computational power, limiting accessibility for users with limited resources.

Some mobile applications have been developed to address the portability issues of phenotyping tools above. For instance, PocketPlant3D [4] enables whole-canopy analysis, and Plant Screen Mobile [17] emphasizes projection-based phenotyping. However, these smartphone applications execute algorithms on users’ devices, but mobile device hardware constraints require specific adaptations, such as model quantization and knowledge distillation, to enable the deployment of sophisticated deep learning models [2]. These requirements often hinder the integration of versatile tools into such platforms.

To address these challenges and meet the need of practical phenotyping, we propose OpenPheno, an open-access, user-friendly, and smartphone-based software platform for instant plant phenotyping. A key feature of OpenPheno is that it interfaces with a WeChat Mini-Program. OpenPheno harnesses this interface to deliver a portable and intuitive user experience for plant phenotyping, with also image processing conducted on remote servers to ensure fast and reliable performance even with large models.

**Fig. 2:**
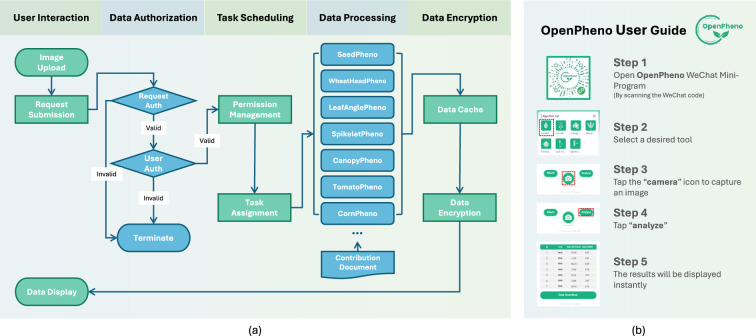
Introduction of OpenPheno software. **a** Pipeline of WeChat Mini-Program. The workflow consists of five stages: user interaction, data authorization, task scheduling, data processing, and data encryption, covering the entire process from image upload to data display. **b** User Guide. Users can analyze phenotypes using OpenPheno in five simple steps

Using OpenPheno is straightforward for users, as illustrated in Fig. 1.

In this paper, we present the workflow and algorithm contribution methods of the OpenPheno platform, and use several phenotyping application cases as examples to illustrate our work.

By integrating advanced computational methods with a mobile and cloud-based infrastructure, OpenPheno aims to democratize plant phenotyping by making advanced tools accessible to a broader community, including scientists, breeders, and developers. The goal of this study is to bridge the gap between high-performance phenotyping algorithms and real-world usage scenarios, especially in resource-limited environments. To this end, we focus on enabling intuitive smartphone-based data acquisition, cloud-side processing of complex models, and an open mechanism for community-driven algorithm contributions. Through this work, we aim to promote broader participation in phenotyping research and empower users across different disciplines to efficiently analyze plant traits in various contexts.

## Methods

### Software structure

The OpenPheno system is built upon a client–server architecture that emphasizes scalability and modularity for phenotypic data analysis. In this architecture, the client handles image acquisition and user interaction, while the server is responsible for algorithmic processing and result delivery. The system is mainly organized into five core components:

- *Client interface* OpenPheno offers a mobile front-end via a WeChat Mini-Program, enabling seamless cross-platform access and providing an intuitive, easy-to-use interface.
- *Algorithm library* Each client-side task maps one-to-one to a corresponding algorithm in the library. The OpenPheno platform currently includes seven phenotyping algorithms—SeedPheno, WheatHeadPheno, LeafAnglePheno, SpikeletPheno, CanopyPheno, TomatoPheno, and CornPheno. Each algorithm is implemented in Python and stored on the server.
- *Application interface (API)* An API facilitates communication between the client and the server. It provides endpoints such as /analyze and /results, which use HTTP POST methods and JSON payloads for data exchange.
- *Task dispatcher* Upon receiving a request, the server identifies the appropriate phenotyping task based on the task identifier and dispatches it to the corresponding model.
- *Model executor* The selected algorithm is executed within an isolated Python environment on the server, ensuring reproducibility and resource encapsulation. This component processes the submitted image and generates phenotypic outputs, such as segmentation masks or quantitative trait measurements.

This modular design ensures a clear separation of responsibilities, simplifies maintenance, and facilitates the integration of additional phenotyping models. Moreover, the use of a request-response communication paradigm supports asynchronous and efficient task execution between the client and server components.

### Software workflow

While the system structure outlines the modular responsibilities, the software workflow emphasizes the runtime execution sequence—from user input to result delivery. The complete pipeline of the OpenPheno software is illustrated in Fig.2a. The workflow comprises two collaborative components: client-side interaction and server-side processing.

***Client-side***

The client provides an intuitive five-step interaction flow, as shown in Fig. 2b. Users begin by launching the OpenPheno WeChat Mini-Program and selecting a desired phenotyping tool. They capture an image using the built-in camera interface and tap the “Analyze” button. The server-side pipeline is triggered transparently, and the results are automatically displayed once processing is complete.

This workflow ensures a seamless user experience while maintaining backend robustness and scalability for real-time analysis.

***Server-side***

The server pipeline comprises five sequential stages:

- *User request handling* Each analysis task is initiated by a client request, triggering server-side execution.
- *Access authorization* The server verifies the legitimacy of the request and user credentials to ensure secure access.
- *Task scheduling* Upon authorization, the request is dispatched to the corresponding algorithm module, as determined by the task identifier.
- *Data processing* The selected model performs image analysis and computes phenotypic outputs.
- *Result packaging and encryption* The final results are encrypted and temporarily cached before being returned to the client.

### Algorithm contribution

**Table 2:** Standard directory structure for algorithm contribution to OpenPheno

File/folder	Description
main.py	Entry point script; defines main_algorithm(image)
weights/	Folder containing model weights
test_samples/	2–5 test images for validation
output/	Directory for saving inference outputs
requirements.txt	Python dependencies with versions
README.md	Description, usage, author info, and environment requirements

A key feature of the OpenPheno platform is its openness. Developers can contribute algorithms, bringing their ideas to life and offering users advanced tools. Approved contributions will be integrated into the OpenPheno algorithm library and made available to all users.

To ensure consistency and compatibility, OpenPheno establishes a standardized contribution workflow, as illustrated by the standard directory structure shown in Table 2. Contributors are required to submit their algorithm in Python, following a unified format that includes the entry-point script (main.py), model weights, test samples, output examples, dependency specifications (requirements.txt), and documentation (README.md). The core function main_algorithm(image) must be defined as the main processing entry.

Submitted packages undergo automated validation, including checks for file structure, code completeness, dependency conflicts, and inference output. Additionally, each algorithm is evaluated using the provided test samples to ensure runtime correctness. Upon approval, the algorithm will be published with a version number and made available to the OpenPheno community. Developers can further maintain and update their algorithms through versioned resubmissions.

### Features

Notable features of OpenPheno include:

- *Zero-cost for users* OpenPheno does not need specialized hardware and software installations, offering a cost-free solution for plant phenotyping users. By leveraging cloud computing, OpenPheno can tackle intensive image processing, allowing users to bypass local computational constraints and access high-performance resources without additional costs.
- *Diversity and openness* OpenPheno supports the image analysis of various plant species. Algorithm developers or researchers can easily deploy their own phenotyping tools and contribute to the community to further enhance the platform diversity.
- *Portability and easy accessibility* OpenPheno is accessible via smartphone through the WeChat Mini-Program. Users can analyze plant traits with OpenPheno in field and get phenotype results in real time. In addition, it provides a user-friendly GUI, designed for users with different backgrounds.
- *Cross-platform* OpenPheno operates seamlessly on both Android and iOS platforms, reducing platform-related barriers and ensuring broad accessibility for all users.

## Results

Several applications are currently deployed on OpenPheno, including SeedPheno, WheatHeadPheno, LeafAnglePheno, SpikeletPheno, CanopyPheno, TomatoPheno, and CornPheno. All applications leverage up-to-date methods to address phenotypic trait analysis across various plant species.

The cloud-based architecture of OpenPheno decouples application performance from device hardware limitations, allowing the integration of more computationally intensive models. As a result, the platform supports a wider range of phenotyping tasks with enhanced accuracy and responsiveness.

An overview of the methods and features of these applications is provided in Table 3. Detailed descriptions are given below.

**Table 3:** Features of applications on the OpenPheno platform

Application	Plant type	Main method	Phenotypic traits	Recommand setup
SeedPheno	All Seeds	YOLO, OTSU	Seed count, aspect ratio, perimeter, etc.	Top-down view, black background, coin for scale
WheatHeadPheno	Wheat	YOLO	Wheat head count, head length/width, yield estimate	Overhead view field image
LeafAnglePheno	Wheat	YOLO	Flag leaf to stem angle	Plain background, top-down wheat placement
SpikeletPheno	Wheat	CBAM-Unet	Spikelet count, spikelet angle, morphology	Side view of ear, black background
CanopyPheno	General	YOLO, K-Net	Canopy area, green ratio, compactness, etc.	Top-down image, dark background preferred
TomatoPheno	Tomato	HSV	Tomato count, diameter, perimeter, area	Top-down view, black/dark background, coin for scale
CornPheno	Corn	PET, K-Means	Kernel count, rows per ear, kernels per row	Horizontal ear view, field or lab

### SeedPheno

**Fig. 3:**
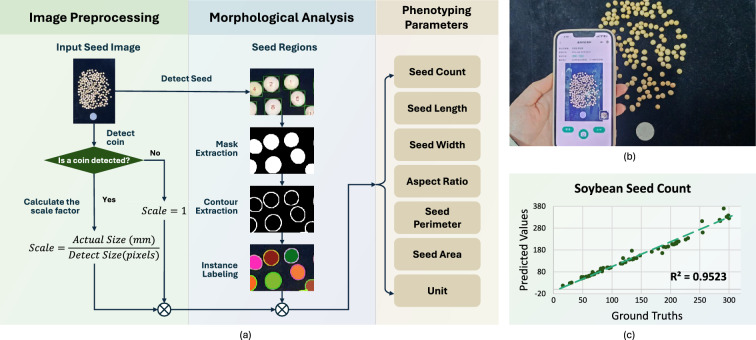
Introduction of SeedPheno. SeedPheno provides functions such as seed counting, seed length measurement, seed width measurement, etc. **a** Technical pipeline of SeedPheno. **b** Seed image capturing. **c** SeedPheno Soundness (Soybean Seed Count)

SeedPheno is an automated seed analysis tool that efficiently processes and analyzes seed images to extract valuable morphological information. As shown in Fig. 3a, it consists of two main stages: image preprocessing and morphological analysis. Initially, coin detection establishes the scale factor, followed by seed detection to identify seed regions. SeedPheno employs You Look Only Once (YOLO) v8 as the object detection model, which is highly effective in recognizing seeds of varying colors and shapes. The training dataset includes a diverse range of seed types, such as corn, soybeans, red beans, green beans, pumpkin seeds, watermelon seeds, and coffee beans. After detecting the seed regions, the system uses the OTSU method to segment the seeds and obtain their mask regions. This segmentation step is essential for accurately extracting the contours and performing instance labeling for the final results. In the morphological analysis stage, SeedPheno extracts various parameters, such as seed count, length, width, perimeter, area, and aspect ratio. Additionally, the system provides unit conversions to ensure the results are easily interpretable and applicable for different purposes.

Using SeedPheno is simple, as shown in Fig. 3b. One is suggested to place a black background and capture seeds from a top-down view. A coin can be placed in the image to offer a reference for estimating the real-world size of the seeds. An $R^2$-based evaluation is shown in Fig. 3c.

### WheatHeadPheno

**Fig. 4:**
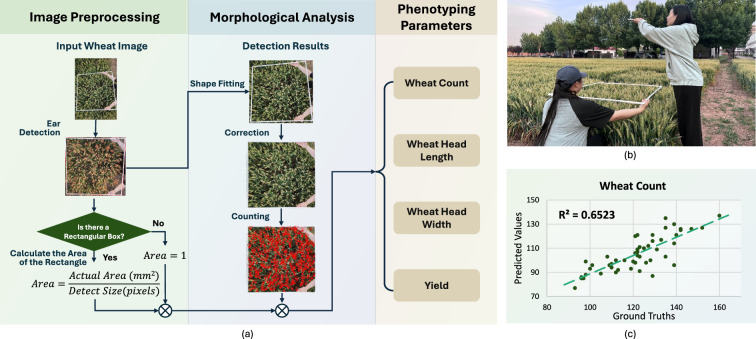
Introduction of WheatHeadPheno. WheatHeadPheno provides wheat head counting and other phenotyping parameters. **a** Technical pipeline of WheatHeadPheno. **b** Wheat image capturing. **c** WheatHeadPheno Soundness (Wheat Count)

WheatHeadPheno is a system developed for in-field mature wheat head analysis. As shown in Fig.4a, it involves two phases: image preprocessing and morphological analysis. The workflow includes ear detection, morphological rectification, and quantitative enumeration from input images. The system measures wheat dimensions (length and width) in rectified regions and performs object enumeration for wheat counts. Upon identifying rectangular markers, standardized verification occurs to estimate acreage yield through spatial calibration. Final outputs include key agronomic parameters: wheat count, head length, head width, and yield.

Figure 4b shows the operational protocol. The system requires dual-user collaboration. One operator marks uniform wheat areas with white rectangles, while another captures top-view images of both markers and wheat for automated analysis. Figure 4c illustrates the $R^2$ evaluation.

### LeafAnglePheno

**Fig. 5:**
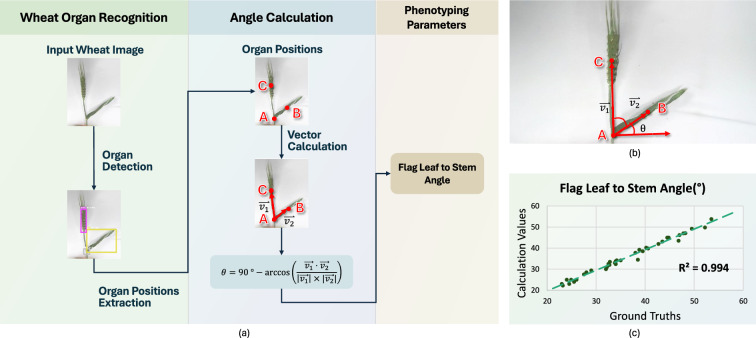
Introduction of LeafAnglePheno. LeafAnglePheno is a tool used for calculating the inclination angle of flag leaves. **a** Technical pipeline of LeafAnglePheno. **b** Wheat image capturing. **c** LeafAnglePheno Soundness (Flag Leaf to Steam Angle)

Precise flag leaf angle detection is vital for analyzing wheat morphology and advancing breeding efforts [23]. LeafAnglePheno is developed to support this task by providing automated angle measurement.

The proposed method has two components: wheat organ recognition and flag leaf angle calculation, as shown in Fig. 5a. In the first stage, wheat images are processed using a module that detects leaves, stems, and spikes. The center coordinates of the bounding boxes are extracted to define three key points: stem base, spike tip, and leaf node (A, B, and C), illustrated in Fig. 5b. In the second stage, vectors between these key points determine the leaf angle.

Figure 5b depicts the image capture setup for LeafAnglePheno, which needs a plain background for accurate wheat detection. An evaluation based on $R^2$ is shown in Fig. 5c.

### SpikeletPheno

**Fig. 6:**
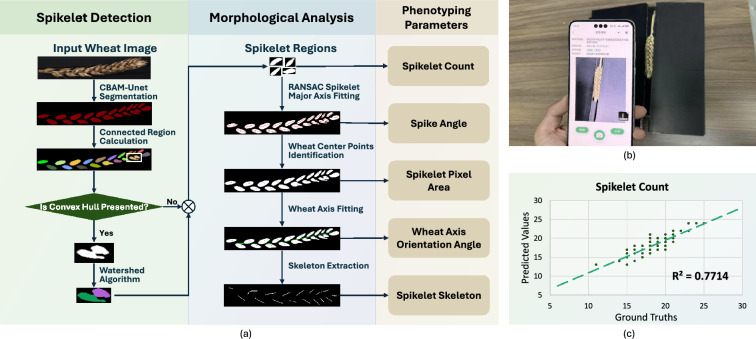
Introduction of SpikeletPheno. SpikeletPheno facilitates spikelet phenotyping tasks. **a** Technical pipeline of SpikeletPheno. **b** Spikelet image capturing. **c** SpikeletPheno Soundness (Spikelet Count)

Accurate identification and morphological analysis of spikelets in mature wheat ears are essential for advancing wheat breeding research [3, 12]. Traditional methods for spikelet recognition and analysis encounter significant challenges, such as adhesion between spikelets and difficulties in morphological characterization. To address these challenges, SpikeletPheno proposes an improved spikelet recognition and morphological analysis method.

The proposed method includes spikelet counting and morphological analysis, as shown in Fig.6a. A wheat ear image is first processed using the CBAM-UNet model, a Convolutional Block Attention Modules (CBAMs) [25] enhanced UNet framework [19], segmenting wheat spikelets from background. Residual adhesion between spikelets is resolved using a convex hull-based approach, distance transformation, and the watershed algorithm, enabling the identification and counting of individual spikelets. Morphological analysis follows, where the RANdom SAmple Consensus (RANSAC) algorithm fits the major axis of each spikelet, and their midpoints are identified as center points. A polynomial regression calculates the wheat ear axis, and finally the Zhang-Suen thinning algorithm [28] extracts the skeleton of the spikelet distribution along the axis.

SpikeletPheno facilitates data collection with minimal equipment: two black, light-absorbing cardboard sheets and a smartphone. Users only need to position the wheat ear between the sheets, ensuring a side-view orientation against a completely black background, and capture images from a top-down perspective using a smartphone with a camera resolution of at least 12 megapixels. An example is shown in Fig. 6b. These images are subsequently processed by the method above. Figure 6c illustrates the $R^2$ evaluation.

### CanopyPheno

**Fig. 7:**
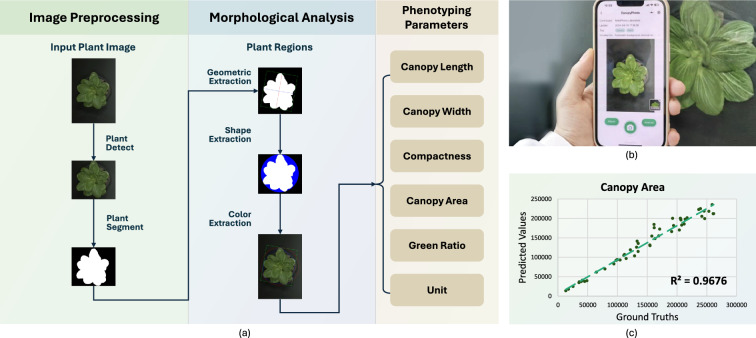
Introduction of CanopyPheno. CanopyPheno provides parameters for plant canopy phenotyping. **a** Technical pipeline of CanopyPheno. **b** Plant canopy image capturing. **c** CanopyPheno Soundness (Canopy Area)

CanopyPheno facilitates plant canopy analysis, with its pipeline illustrated in Fig. 7a. First, the input plant image undergoes detection and segmentation to extract regions. Specifically, CanopyPheno employs YOLO v8 as the object detection model to identify plant canopy regions. Then, K-Net is used as the semantic segmentation model to extract the mask of the plant canopy. The training dataset includes a large number of canopy images from various green plants, such as rice, wheat, soybeans, corn, lettuce, and tobacco. Then, shape and color extraction analyze the plant’s characteristics. Finally, parameters such as canopy length, width, compactness, area, green ratio, and other metrics are computed.

Using CanopyPheno is also straightforward, as illustrated in Fig. 7b. Simply take a top-down photo of the target plant. A dark-colored background can improve recognition results. Figure 7c displays the evaluation results.

### TomatoPheno

**Fig. 8:**
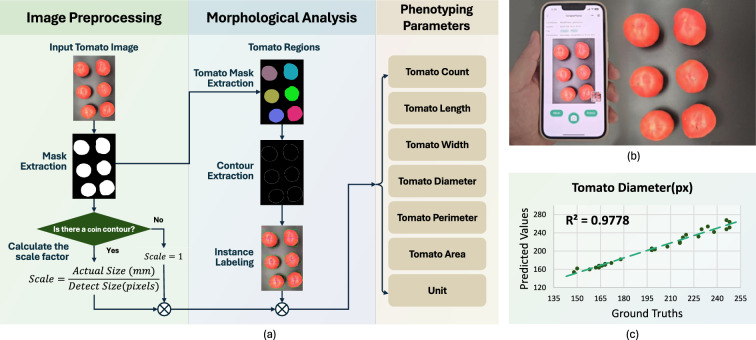
Introduction of TomatoPheno. TomatoPheno provides parameters for tomato phenotyping. **a** Technical pipeline of TomatoPheno. **b** Tomato image capturing. **c** TomatoPheno Soundness (Tomato Diameter)

The pipeline of TomatoPheno for automated tomato fruit analysis is depicted in Fig. 8a. First, the input image is converted to the HSV color space to maximize the color difference between tomatoes and the background. Then, the system sets upper and lower bounds for each channel (H, S, V) to extract the tomato foreground regions. After that, mask extraction is performed to identify tomato regions, followed by contour extraction and instance labeling for segmentation. If a coin is detected, a contour check determines the scale factor, incorporated into the measurements. The system computes key parameters: tomato count, width, diameter, perimeter, area, and unit conversions.

Using TomatoPheno is simple, as shown in Fig. 8b. Capture a top-down image of the tomatoes, preferably against a dark-colored background to enhance detection accuracy. Figure 8c provides an overview of the $R^2$ evaluation.

### CornPheno

**Fig. 9:**
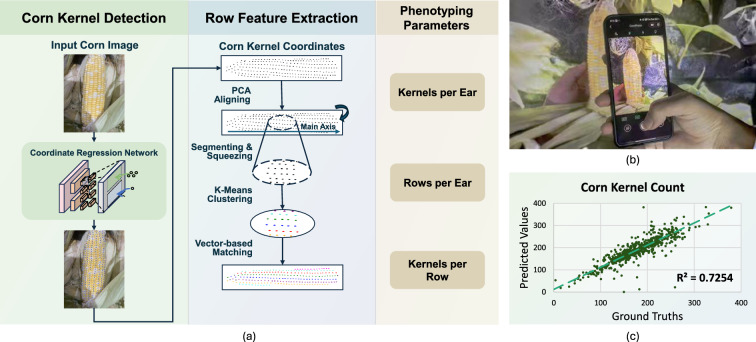
Introduction of CornPheno. CornPheno is a corn kernel phenotyping tool that operates in both lab and in-field environments. **a** Technical pipeline of CornPheno. **b** Corn image capturing. **c** CornPheno Soundness (corn kernel count)

Accurate and efficient phenotyping of corn is crucial for breeding and agricultural research, in which corn kernel count plays a important role. Various techniques are presented but face limitations in field environments [8, 16, 22]. To overcome these problems, the CornPheno tool has been developed to streamline the corn phenotyping process, offering more precise and scalable solutions for trait analysis in corn breeding.

The pipeline of CornPheno is illustrated in Fig. 9a, consisting of two main stages: corn kernel detection and row feature extraction. In the first stage, corn kernel detection is performed using a coordinate regression network, Point quEry Transformer (PET) [13]. The input image undergoes processing, resulting in the final corn kernel detection output, which is then passed to the next stage. In the second stage of CornPheno, additional features are extracted from the labeled corn kernels. Principal Component Analysis (PCA) and K-means are used for kernels segmentation, clustering, and matching. Ultimately, the total corn kernel count, row count, and kernels per row are obtained.

In contrast to traditional machine-based corn seed phenotyping methods, thanks to the feature of OpenPheno, CornPheno introduces a smartphone-based solution that significantly reduces operational costs and enables image acquisition in open-field settings. The cloud-based, open-access architecture further supports the execution of complex deep learning algorithms without local computational burden, making it possible to perform advanced phenotypic analysis instantly. This is the first system to enable real-time phenotyping of corn traits in open environments through a fully mobile and accessible platform, representing a notable step forward in making high-quality agricultural tools more widely available.

CornPheno is robust, accepting pictures of corn captured in both lab and in-field environments, as shown in Fig. 9b. The $R^2$ evaluation is depicted in Fig. 9c.

## Discussion

Rapid advancements in modern computer vision methods are transforming traditional breeding by making standardized data acquisition more efficient and accessible [26, 27]. OpenPheno is pivotal in this shift, providing a user-friendly, smartphone-based platform for real-time plant phenotyping. Users can quickly extract standardized phenotypic data, such as seed size, plant height, and leaf angle, from images. These data are vital for further AI-driven breeding algorithms, which need precise inputs to identify genotype-phenotype relationships [6]. By enabling faster and more accurate trait analysis, OpenPheno accelerates the selection of traits like higher yield or disease resistance, enhancing breeding program efficiency.

Smartphone-based agricultural tools are gaining popularity due to mobile device and internet accessibility. They offer farmers and researchers portable, cost-effective solutions for data collection and analysis, making advanced agricultural technologies more accessible [4, 17, 21].

Although the current implementation of OpenPheno relies on the WeChat Mini-Program ecosystem, which is indeed a potential limitation of our current implementation, we also note that WeChat provides a lot of benefits: it has cross-platform capability and is free to access. For users who already use WeChat, no additional installation is required; for those who do not, installing WeChat is no more burdensome than downloading any other mobile application.

OpenPheno enhances this by integrating advanced computer vision methods, especially some AI-based phenotyping tools with smartphones. Users can capture and analyze plant images directly from their devices, streamlining data collection and making breeding tools accessible to non-experts. This democratization of technology not only enhances precision agriculture but also promotes the adoption of digital farming practices, especially in rural areas with limited resources. By merging mobile technology with modern computational algorithms, OpenPheno supports more efficient, scalable, and inclusive agricultural research and breeding.

## Conclusions

In conclusion, OpenPheno offers a convenient and efficient platform for plant phenotyping. It overcomes limitations of existing tools, such as high costs and lack of immediacy, while lowering entry barriers through an intuitive interface and strong technical support. This accessibility benefits researchers without an information science background. OpenPheno also explores mobile devices’ potential in plant phenotyping, fostering a more open, collaborative, and user-friendly research ecosystem.

### Availability and requirements

- **Project name**: OpenPheno
- **Project home page**: https://github.com/openpheno/OpenPheno
- **Operating system(s)**: iOS, Android (The WeChat Mini-Program runs on the WeChat client)
- **Programming language**: JavaScript/WXML/WXSS (frontend WeChat Mini-Program development), Python (backend and algorithm development)
- **Other requirements**: Python 3.6 or higher
- **License**: MIT License

## Data Availability

Evaluation sample data used for algorithm validation and demonstration has been made publicly available at out GitHub repository: https://github.com/openpheno/OpenPheno/tree/main/dataset.
